# Comparative evaluation of the accuracy of skin tests and measurement of mite-specific IgE in patients with allergic rhinitis

**DOI:** 10.1186/1939-4551-8-S1-A146

**Published:** 2015-04-08

**Authors:** Helen Cruz Rito, Norma Rubini, Albertina Varandas Capelo, Eliane Miranda Da Silva, Fernando Samuel Sion, Carlos Alberto Morais De Sa

**Affiliations:** 1 Federal University of the State of Rio De Janeiro, Brazil

## Background

The diagnostic investigation of sensitization to dust mites is performed through measurement of specific IgE and cutaneous immediate hypersensitivity tests. The general aim of this study was to comparatively evaluate the diagnostic accuracy of immediate hypersensitivity skin tests and specific IgE for Dermatophagoides pteronyssinus (Dp) and Blomia tropicalis in patients with allergic rhinitis.

## Methods

A retrospective study evaluating patients between 5-65 years of age, of both sexes, with a clinical diagnosis of allergic rhinitis, was performed. The control group consisted of staff and students of the institution, aged 18-65 years, of both sexes, and no prior or current history of allergic rhinitis, asthma or atopic dermatitis. The measurement of specific IgE for Dp and Bt was performed using the ImmunoCAP and in the immediate hypersensitivity to Dp and Bt skin tests extracts from the FDA Allergenic were used.

## Results

We evaluated 42 patients with allergic rhinitis and included 22 patients in the control group. We observed 90% sensitivity for Dp skin tests and 66.7% for measurement of specific IgE to Dp (p = 0.0001); 84% for Bt skin tests, and 61.9% for IgE specific for Bt (p = 0.0004). The specificity was 91% for Dp and Bt skin tests and 86% for specific Bt and Dp IgE (p> 0.05). The positive predictive value (PPV) was 95% for Dp skin tests, 94% for BT skin tests and 90% for serum specific IgE for both Dp and BT (p> 0.05). The negative predictive value (NPV) was 83% for Dp skin tests and 58% for the measurement of specific IgE for Dp (p = 0.0001); 77% for Bt skin tests and 54% for serum specific IgE for Bt (p = 0.001).

## Conclusions

In the studied population, there was a higher sensitivity and better NPV of skin tests for Dp and Bt compared to the dosage of specific IgE. These results reinforce the usefulness of skin tests for immediate hypersensitivity to mites in clinical practice.

